# Dual Acting Carbon Monoxide Releasing Molecules and Carbonic Anhydrase Inhibitors Differentially Modulate Inflammation in Human Tenocytes

**DOI:** 10.3390/biomedicines9020141

**Published:** 2021-02-01

**Authors:** Marialucia Gallorini, Anna C. Berardi, Alessia Ricci, Cristina Antonetti Lamorgese Passeri, Susi Zara, Francesco Oliva, Amelia Cataldi, Fabrizio Carta, Simone Carradori

**Affiliations:** 1 Department of Pharmacy, University “G. d’Annunzio” of Chieti-Pescara, Via dei Vestini 31, 66100 Chieti, Italy; alessia.ricci@unich.it (A.R.); susi.zara@unich.it (S.Z.); cataldi@unich.it (A.C.); simone.carradori@unich.it (S.C.); 2 Laboratory of Stem Cells, U.O.C. of Immunohematology and Transfusion Medicine, Santo Spirito Hospital, 65122 Pescara, Italy; cristinaalp94@gmail.com; 3 Department of Musculoskeletal Disorders, Faculty of Medicine and Surgery, University of Salerno, 84081 Baronissi, Italy; foliva@unisa.it; 4 Department of NEUROFARBA, University of Florence, Section of Pharmaceutical and Nutraceutical Sciences, Polo Scientifico, Via U. Schiff 6, 50019 Firenze, Italy; fabrizio.carta@unifi.it

**Keywords:** tendons, inflammation, rotator cuff, Nrf2, iNOS, carbonic anhydrase, CORM

## Abstract

Sustained oxidative stress and inflammation have been reported as the major factors responsible for the failure of tendon healing during rotator cuff tears (RCTs) and rotator cuff disease (RCD). Although, their therapeutic management remains still challenging. Carbonic anhydrases (CAs) are involved in many pathological conditions, and the overexpression of both CA9 and 12 in inflamed joints has been recently reported. Consequently, a selective CA9/12 inhibition could be a feasible strategy for improving tendon recovery after injury. In addition, since carbon monoxide (CO) has been proven to have an important role in modulating inflammation, CO releasing molecules (CORMs) can be also potentially suitable compounds. The present study aims at evaluating five newly synthesized dual-mode acting CA inhibitors (CAIs)-CORMs compounds, belonging to two chemical scaffolds, on tendon-derived human primary cells under H_2_O_2_ stimulation in comparison with Meloxicam. Our results show that compounds **2** and **7** are the most promising of the series in counteracting oxidative stress-induced cytotoxicity and display a better profile in terms of enhanced viability, decreased LDH release, and augmented tenocyte proliferation compared to Meloxicam. Moreover, compound **7**, as a potent superoxide scavenger, exerts its action inhibiting NF-ĸB translocation and downregulating iNOS, whereas compound **2** is more effective in increasing collagen I deposition. Taken together, our data highlight a potential role of CA in RCTs and RCD and the prospective effectiveness of compounds acting as CAI-CORM during inflammation.

## 1. Introduction

Degenerative non-traumatic rotator cuff tears (RCTs) and rotator cuff disease (RCD) are a common cause of chronic shoulder pain and disability. Their incidence increases with age and affects 30 to 50% of individuals starting from the third decade of life, reaching more than 50% above the fifth one [1]. Intrinsic factors (age or gender), extrinsic factors (sports activity or occupational exposure), and biological factors were identified in the onset and progression of RCTs and RCD [2]. Our group has reported evidence of a significant change in the modulation of pro-inflammatory cytokine secretion and related molecular cell signaling during rotator cuff tendinopathies [3,4]. In these investigations, it has been highlighted that tendon-derived cells escape from oxidative stress and consequently from apoptosis by reducing prostaglandin (PGE2) secretion [4] and by activating the nuclear factor erythroid 2-related factor (Nrf2)-related pathways [5].

Many studies suggest that treatment strategies able to counteract inflammation and improve function would be a potential benefit for RC treatment. To date, there is no established therapeutic strategy in RCTs and RCD due to a lack of evidence about their efficacy. The benefits of non-steroidal anti-inflammatory drugs (NSAIDs) in relieving pain in the acute phase are broadly accepted but their use in chronic tendon-related diseases is controversial [6].

Under oxidative stress and inflammation, Nrf2 directly transcribes for heme oxygenase-1 (HO-1), which produces carbonic monoxide (CO) along with ferrous iron and biliverdin as a crucial cellular stress response that repairs damage and restores cellular homeostasis [7]. The therapeutic significance of a controlled carbon monoxide (CO) delivery at low doses has recently gained reliability being sustained by a broad amount of literature [8,9]. It has been reported that CO administered at low doses can mimic many of the beneficial effects exerted by HO-1 in several experimental models such as inflammatory conditions [10]. In this context, CO releasing molecules (CORMs) are attracting enormous interest. Berrino and colleagues [11] reported a series of small-molecule hybrids consisting of a carbonic anhydrase inhibitor (CAI) linked to a CORM tail section (CAI–CORM hybrids) with properties for the management of rheumatoid arthritis. The metalloenzyme carbonic anhydrase (CA) is ubiquitously expressed in tissues, playing a crucial role in pH regulation, but also in several metabolic pathways such as lipogenesis, gluconeogenesis, and ureagenesis [12]. Carbonic anhydrase type IX (CA9) is known to be expressed in the cartilage, tendons, ligaments, and striated muscle [13]. Notably, CA9 has been reported as a potential marker of mechanical stress of joints, ligaments, and tendons during fetal development [14]. Furthermore, an overexpression of the isoforms 9 and 12 was revealed in the inflamed synovium [15] and CA9 has been linked to the inhibition of cell migration by its interaction with several ion transporters and exchangers at the edge of the damaged site [16]. Consequently, a selective CA9/12 inhibition as well as the administration of external CO by means of CORMs could be a feasible strategy for improving tendon recovery after injury.

**Figure 1:**
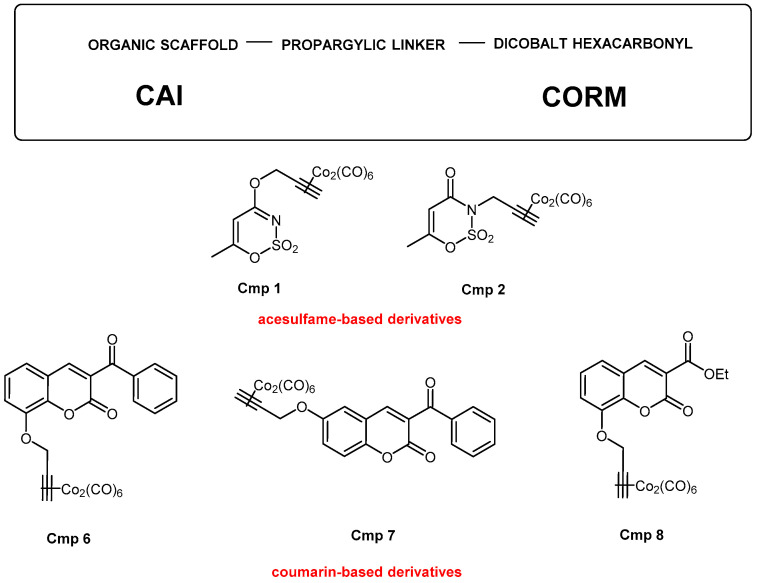
Structures and chemical scaffolds of the five dual carbonic anhydrase inhibitor-carbon monoxide releasing molecule (CAI-CORM) hybrids selected for this study.

In this light, five CAI-CORM hybrids were selected from a series of ten CORMs, already characterized and biologically evaluated in a cell model of inflammation established by lipopolysaccharide (LPS) stimulation on mouse macrophages [17]. These five compounds are characterized by an organic portion belonging to two different chemical scaffolds well-known as selective inhibitors of CA9/12 isoforms, namely acesulfame [18] and coumarin derivatives [19], whereas the CO-releasing group is represented by the dicobalt hexacarbonyl. These two moieties are connected by a propargylic linker as reported in Figure 1. The five compounds, namely compounds **1**, **2**, **6**, **7**, and **8** (Cmp **1–8**) were here administered on rotator cuff-derived human tenocytes from three donors under hydrogen peroxide (H_2_O_2_) stimulation. In parallel, the broadly known NSAID Meloxicam was tested as a term of comparison, being a well-known selective cyclo-oxygenase (COX)-2 inhibitor and thus a potent anti-inflammatory compound [20] The present work aims at highlighting a possible involvement of CA during RCTs and RCD and the effectiveness of compounds acting as CAIs and CO releasers in the inflamed tendon.

## 2. Materials and Methods

### 2.1. Synthesis of CAI-CORM Hybrids

The five CAI-CORM hybrids have been synthesized, purified, and characterized as reported in [17]. Compounds were stored at −20 °C till the biological evaluation.

### 2.2. Cell Culture

Human tenocytes isolated from rotator cuff tendons, obtained from the same 10 donors after their informed consent, described in our previous work [3,4,5,21,22], and cryopreserved in vials in liquid nitrogen, were used. Biopsies were harvested from the healthy tissue close to the surgical site. Samples were anonymized after surgical procedures and delivered unlabeled to the research laboratory. The cell isolation protocol was extensively described previously [3]. Briefly, tendons were washed several times with PBS with 1% penicillin/streptomycin, carefully dissected in small pieces and mechanically disaggregated with the aid of fine watchmaker forceps. Finally, tissue pieces were immediately placed on Petri dishes of 60 mm in diameter (Greiner CELLSTAR dish, Sigma-Aldrich, Saint Louis, MO, USA), containing 5 mL of alpha-MEM supplemented with 20% of heat-inactivated fetal calf serum (FCS), 1% L-glutamine, and 1% penicillin/streptomycin (Gibco, Invitrogen, Life Technologies, Carlsbad, CA, USA) at 37 °C and 5% CO_2_, changing the medium every 2–3 days. Tenocytes were then harvested by StemPro Accutase (Life technologies Carlsbad, CA, USA), and centrifugated at 1500 rpm for 5 min when the cells migrated out of tendon pieces and reached 60–80% of confluence (19 day). For the present work, cells at passage 0 from donor 1 (D1), donor 2 (D2), and donor 3 (D3) were thawed out and promptly cultured to avoid phenotype changes caused by later passages. Tendon-derived cells were maintained in alpha-MEM (EuroClone, Milan, Italy) supplemented with 10% heat-inactivated FBS (Gibco, ThermoFisher Scientific, Waltham, MA USA) and 1% penicillin/streptomycin (EuroClone, Milan, Italy) at 37 °C and 5% CO_2_ and used up to passage 5.

### 2.3. Cell Metabolic Activity

Cells were seeded in 96 well plates (0.5 × 10^4^/well) (ThermoFisher Scientific, Waltham, MA USA) and let them adhere overnight at 37 °C and 5% CO_2_. In a first set of experiments, tendon-derived cells were exposed to loading concentrations of substances alone, for instance, Meloxicam (sodium salt hydrate, Sigma Aldrich, Saint Louis, MO, USA) and CAI-CORM hybrids (compounds **1**, **2**, **6**, **7**, and **8** in the range 0–400 µM) for 24 and 72 h, as well as hydrogen peroxide (0–800 µM, stock solution 30%) (Sigma Aldrich, MA, USA) for 3 and 24 h to obtain dose-response curves. Meloxicam and CAI-CORM compounds were firstly dissolved in DMSO to obtain a 200 mM stock solution and they were then diluted in complete alpha-MEM (DMSO final concentration = 0.2%) for further analyses. In a second set of experiments, cultures were pre-incubated with 100 or 200 µM H_2_O_2_ for 3 h. After that, the pre-incubation medium was discarded and replaced with a fresh one containing H_2_O_2_ and Meloxicam or the proper CAI-CORM hybrid.

At the established time points (24 and 72 h), the incubation medium was harvested for further analyses and complete alpha-MEM containing 0.5 mg/mL MTT (3-[4,5-dimethylthiazol-2-yl]-2,5-diphenyl tetrazolium bromide) (Sigma-Aldrich, St. Louis, MO, USA) was added to each well. Cells were afterwards incubated for 5 h at 37 °C. After having discarded the MTT medium, the same volume of DMSO was added to each well and samples were incubated for 20 min at 37 °C and afterwards gently shaken at room temperature for additional 10 min. The absorbance was measured at 540 nm using a spectrophotometer (Multiscan GO, Thermo Fisher Scientific, Waltham, MA, USA). The percentage of metabolically active cells was calculated setting the pure untreated control (only complete alpha-MEM for the H_2_O_2_ dose-response curve) or the untreated control containing only DMSO 0.2% (0 µM) as 100%.

### 2.4. Cytotoxicity Assay

The release of LDH in cell supernatants was quantified by the CytoTox 96^®^ non-radioactive assay (Promega Corporation, Fitchburg, WI, USA) to assess cytotoxicity occurrence after 24 h. Cell supernatants analyzed were the ones collected from cultures used for the MTT assay. The percentage of LDH released from tendon-derived cells was therefore normalized on the OD values generated from the metabolic activity assay. The CytoTox 96^®^ non-radioactive kit measures the amount of LDH released upon cell lysis using a 30-min coupled enzymatic assay. The assay was carried out as previously reported [4].

### 2.5. Quantification of Protein

After having treated cells (1.5 × 10^4^) in 6 well plates (ThermoFisher Scientific, Waltham, MA USA) for 24 h as previously described for the cytotoxicity assay, cells were trypsinized, centrifuged (1200 rpm), and collected in cold PBS. Cell pellets were lysed and proteins were extracted and quantified by the bicinchoninic acid (BCA) assay as reported elsewhere [23].

### 2.6. Hematoxylin/Eosin Staining

Tendon-derived cells were seeded (5 x 10^3^ cells/well) in well glasses (Millicell^®^ EZ slide, Merck Millipore, Burlington, MA, USA). After 24 h from seeding, tenocytes were pre-incubated with H_2_O_2_ 100 µM for 3 h and afterwards exposed to compounds **2** and **7** at 25 µM for an additional 24 h. Next, supernatants were removed and cells were washed twice with PBS with calcium and magnesium (Euro Clone, Milan, Italy). Tenocytes were fixed in the presence of *p*-formaldehyde 4% and stained with hematoxylin/eosin as reported elsewhere [22].

### 2.7. Cell Cycle Analysis

Aiming to measure the proliferation rate of tendon-derived cells exposed to loading concentrations of Meloxicam and CAI-CORM hybrids, the cell cycle progression was assessed by flow cytometry. Cells were seeded (1.5 × 10^4^/well) in a 6-well tissue culture-treated plate (ThermoFisher Scientific, Waltham, MA, USA) and let them adhere for 24 h. Then, growth medium was removed and replaced with fresh medium mixed with compounds (0–100 µM). After the exposure time (72 h), cells were washed twice with PBS without calcium and magnesium and harvested with Trypsin EDTA 1X (all purchased by EuroClone S.p.a., Milan, Italy) and counted by means of Trypan blue exclusion test (Sigma Aldrich, Milan, Italy). Approximately 5 × 10^5^ cells/experimental condition were fixed with cold ethanol 70% *v/v* and kept overnight at 4 °C. Cells were then suspended in the staining solution made by PBS without calcium and magnesium, 1 mg/mL propidium iodide (PI) (final concentration 10 mg/mL) and 10 mg/mL RNAse (final concentration 100 mg/mL) and kept overnight at 4 °C in the dark. Cell cycle profiles (1 × 10^4^ events/sample) were finally analyzed with a CytoFLEX flow cytometer (Beckman Coulter, Indianapolis, IN, USA) and data were quantified using the ModFit LT™ software (De Novo Software, Glendale, CA, USA).

### 2.8. Collagen Type I Secretion

Absolute amounts of collagen type I secreted in supernatants harvested from 6 well plates were detected by human collagen type 1 ELISA kit (Cosmo Bio Co., Ltd., Tokyo, Japan; cat. no. ACE-EC1-E105-EX). Samples were pipetted into suitably coated wells as described elsewhere [23]. The concentration of collagen type I (μg/mL) was calculated using a standard curve generated with specific standards provided by the manufacturers by means of the Prism 5.0 software (GraphPad, San Diego, CA, USA).

### 2.9. Detection of Mitochondrial Superoxide Anions by Flow Cytometry

The intracellular generation of mitochondrial superoxide anions was determined using the oxidation-sensitive fluorescent probe MitoSOX™ (MitoSOX™ Red Mitochondrial Superoxide Indicator, Invitrogen, ThermoFisher Scientific, Waltham, MA, USA). Briefly, after having treated cultures as described above, cells were incubated with MitoSOX Red at a final concentration of 1 µM (stock solution in DMSO 1 mM) in the exposure medium for 30 min at 37 °C and 5% CO_2_ in the dark. Next, tendon-derived cells were trypsinized, harvested, and washed as described for cell cycle analysis. After the final washing in PBS, 300 µL of PBS were added and fluorescent cell suspensions were analyzed using a CytoFLEX flow cytometer (Beckman Coulter, Indianapolis, IN, USA) equipped with a 488 nm laser with the fluorescence channel FL-2/PE in a linear mode. Relative fluorescence emissions of gated cells by means of their forward and side scatter properties (FSC/SSC) were analyzed with the CytExpert software (Beckman Coulter, Indianapolis, IN, USA) and they were expressed as mean fluorescence intensity ratios on the unstained control.

### 2.10. Protein Expression by Western Blot Analysis

Tenocytes were lysed, and 15 µg of each sample was separated on a 4–20% SDS-PAGE Gel by electrophoresis (ExpressPlus. 10 × 8, GenScript Biotech Corporation, Nanjing, China). After that, samples were transferred to nitrocellulose membranes, as already described [22]. Next, membranes were incubated in the presence of mouse monoclonal anti-β-actin (1:10,000) (Sigma-Aldrich, St. Louis, MO, USA), mouse monoclonal anti-iNOS (1:200), rabbit polyclonal anti-Nrf2 (1:750) (all from Santa Cruz Biotechnology, Santa Cruz, CA, USA), and rabbit monoclonal anti-h-TERT (1:500) (ThermoFisher Scientific, Waltham, MA, USA). After an overnight incubation at 4 °C with primary antibodies under gentle shaking, membranes were then probed with specific IgG horseradish peroxidase (HRP)-conjugated secondary antibodies and bands were identified by chemiluminescence as previously described [22]. At least three independent experiments were performed for each protein. Results are expressed as mean values ± standard deviation (S.D.) of normalized densitometric values on the loading control (β-actin).

### 2.11. Immunofluorescence Staining of NF-kB

Tenocytes were seeded in 4-well chamber slides at 2 × 10^4^/well (NuncTM Lab-TekTM II Chamber SlideTM, Thermo Fisher Scientific, Waltham, MA, USA) in triplicates and cultured as previously described in this section. After 24 h of exposure, cell supernatants were discarded and cultures were prepared for the immunofluorescence staining of NF-kB (1:100, mouse monoclonal, Santa Cruz Biotechnology, Santa Cruz, CA, USA) as previously reported [5].

### 2.12. Statistics

Statistics were performed using one-way analysis of variance (ANOVA) followed by Tukey’s multiple comparison test by means of the Prism 5.0 software (GraphPad, San Diego, CA, USA). Results are the mean values ± standard deviations. Values of *p* ≤ 0.05 were considered statistically significant.

## 3. Results and Discussion

Today, the management of musculoskeletal pathologies represents a major societal burden. Among them, rotator cuff tears (RCTs) and rotator cuff disease (RCD) are two of the most frequent and disabling, reaching percentages between 30 to 70% in the middle-aged population [1]. To date, the use of NSAIDs in chronic tendinopathies is open to discussion, despite their use as painkillers in the acute phase being well documented [6]. Thus, an established and accounted therapeutic strategy for RCTs and RCD is still needed. In this work, dual-acting CAI-CORM hybrids (Figure 1), obtained as previously reported [11,17], were tested on human tenocytes from the rotator cuff of three different donors under H_2_O_2_ stimulation in parallel with Meloxicam. The present work was designed with the aim of demonstrating that the combined properties of both moieties can synergistically counteract inflammation.

### 3.1. Establishment of the Inflammatory Cell Model and Preliminary Investigations

With the intention of establishing sub-toxic oxidative stress conditions in vitro, tenocytes were firstly exposed to loading concentration of H_2_O_2_ (0–800 µM) for 3 and 24 h (SI-Figure S1A). The best H_2_O_2_ concentrations (100 and 200 µM) and incubation-time (3 h), capable of stimulating oxidative stress-related decrease of metabolic activity, were chosen accordingly to MTT results and used for further analyses. Briefly, 100 µM H_2_O_2_ is significantly effective on decreasing cell viability already after short exposures (76.83% at 3 h) and 200 µM H_2_O_2_ shows a dramatic cytotoxicity over the time (around 35% of metabolic activity for both the exposure times). In parallel, increasing concentrations of Meloxicam (SI-Figure S1B) and of all the selected CAI-CORM hybrids (SI-Figure S2) were tested on tenocytes without H_2_O_2_ for showing their behavior under non-oxidative stress conditions. As expected for Meloxicam alone, it shows only a slight and non-significant effect on cell metabolic activity up to 200 µM with respect to DMSO alone (vehicle). Contrariwise, the five CAI-CORM hybrids exert a notable influence on tenocyte metabolic activity and the effect is dependent on the single donor. In details, for D1 Cmp **7** raises cell percentages from 117.75% (6.25 µM) up to 145.64% at the highest concentration after 72 h. On the other hand, Cmp **2** and **6** seem more effective at 24 h on D2. This trend is confirmed after 72 h, with a dose-dependent increase in cell metabolic activity with Cmp **2** and Cmp **6** (around 169% at 200 µM for both). Surprisingly, the same increase is registered with Cmp **7**. Again, Cmp **2**, **6**, and **7** disclose percentages of metabolic activity higher than the ones of controls both at 24 and 72 h in the presence of cells from donor 3 (D3). After 72 h, the increase is clearly dose-dependent, mainly for Cmp **6** and **7** (SI-Figure S2).

Even if Cmp **6** shows good percentages on two different donors, only Cmp **2** and **7** were chosen for further analyses. Firstly, Cmp **6** and **7** are both coumarins and structural isomers, thus presumably owning similar biological activity and molecular mechanism of action. Secondly, Cmp **7** displays a reasonable *K*_i_ on the human CA9 isoform (8.11 µM), while Cmp **6** inhibits only the isoform 12. Thirdly, Cmp **2** is ineffective on CA9 but it revealed a good biological activity on LPS-stimulated murine macrophages, plausibly referable to its CO release profile [17]. Lastly, both compounds were characterized by a slow and medium-efficiency release of CO up to five hours ([MbCO] of 3 µM for Cmp **2** and [MbCO] of 6 µM for Cmp **7**), which seems to be an important requisite to ensure a modulation of the inflammatory process in mouse macrophages [17].

The role of the macrophage is an area of emerging interest in tendinopathy and tendon healing [24,25]. Indeed, inflammation appears to be driven by a high number of infiltrating macrophages at the inflamed tendon site [24]. Furthermore, diseased tendons from patients with tendinopathy show an abundance of CD14^+^ and CD68^+^ activated cells [25]. Thus, Cmp **2** and **7** could be suitable compounds for this purpose, being active both on LPS-stimulated macrophages and on inflamed tenocytes.

### 3.2. Cell Metabolic Activity of Tenocytes under H_2_O_2_ Stimulation in the Presence of Compounds

**Figure 2:**
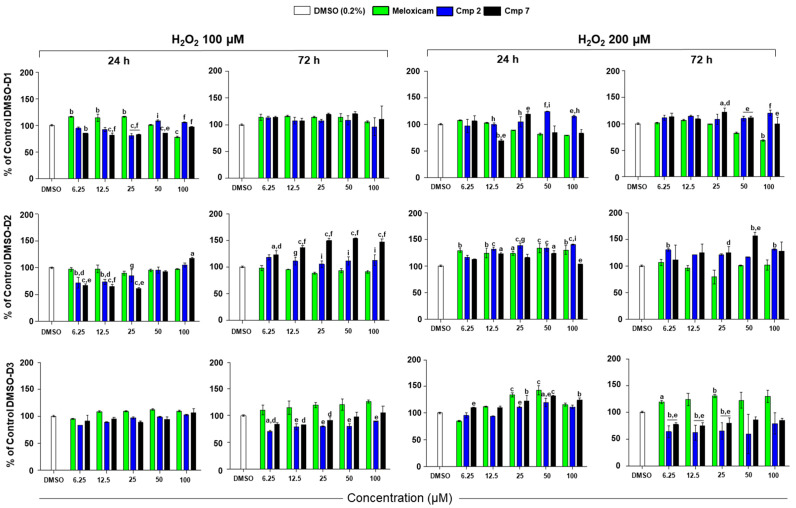
Metabolic activity of tendon-derived cells pre-incubated with hydrogen peroxide (H_2_O_2_) in the presence of Meloxicam and Cmp **2** and Cmp **7**. Data are reported as means ± standard deviations of independent experiments with the three donors separately (n = 9). D1 = donor 1; D2 = donor 2; D3 = donor 3. Bar graphs show the percentages of cell metabolic activity of tenocytes pre-incubated with H_2_O_2_ 100 (**A**) and 200 µM (**B**) for 3 h and afterwards exposed to loading concentrations of Meloxicam, Cmp **2**, and Cmp **7** for 24 and 72 h. Cells treated with DMSO alone (vehicle) were set as control (100% of cell metabolic activity). DMSO = cultures pre-incubated with H_2_O_2_ and treated with dimethyl sulfoxide (DMSO) 0.2%. a = *p* < 0.01; b = *p* < 0.001; c = *p* < 0.0001 between samples and DMSO alone; d = *p* < 0.01; e = *p* < 0.001; f = *p* < 0.0001 between samples and Meloxicam at the same concentration; g = *p* < 0.01; h = *p* < 0.001; i = *p* < 0.0001 between Cmp **2** and Cmp **7** at the same concentration.

After having tested Meloxicam and compounds **2** and **7** alone up to 200 µM, the 3 h pre-incubation with 100–200 µM H_2_O_2_ was performed to mimic inflammation. The maximum dose of compounds was established at 100 µM, being the effects very similar to the ones occurring at 200 µM. Again, cell metabolic responses to CAI-CORM hybrids and Meloxicam show differences depending on the three donors and on the H_2_O_2_ concentration (Figure 2).

For instance, Cmp **2** is the best compound in the counteraction of the H_2_O_2_ stimulation compared to Meloxicam in cells obtained from D1. Despite initially not effective, cell metabolic activity is assessed at 108.7% with Cmp **2** 50 µM, while is around 100% with Meloxicam at the same dose after 24 h. In the presence of 200 µM H_2_O_2_, cell metabolic activity is even more significantly increased with Cmp **2** at 24 h, being dose-dependently raised up to 124.3% with 50 µM compared to 81.7% of Meloxicam at the same concentration. After 72 h, a peak is registered also for Cmp **7** at 25 µM (122.5%). Confirming MTT data obtained from the administration of Cmp **7** alone in tenocytes from D2, cell metabolic activity is extremely high when this hybrid is added to cultures after a H_2_O_2_ stimulation, mainly at 72 h. At that time point, cell percentages arise in a dose-dependent manner, from 122.7% at 6.25 µM up to 153.9% at 50 µM with 100 µM H_2_O_2_, whereas Meloxicam failed in counteracting oxidative stress being percentages registered below 100%. The same trend is maintained also with 200 µM H_2_O_2_. Compound **2** can counteract H_2_O_2_ effects but in a lesser extent. Not in line with results on the other two donors, Meloxicam is the best compound in ameliorating cell metabolic activity of tenocytes from D3 after a 3 h H_2_O_2_ pre-incubation.

### 3.3. Counteraction of H_2_O_2_-Induced Cytotoxicity

**Figure 3:**
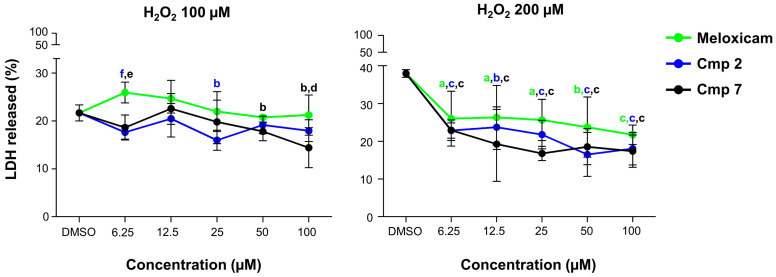
Lactate dehydrogenase (LDH) released from tendon-derived cells pre-incubated with hydrogen peroxide (H_2_O_2_) in the presence of Meloxicam and Cmp **2** and Cmp **7**. Data are reported as means ± standard deviations of independent experiments with the three donors pooled (n = 9). Trend lines show the percentage of LDH released from tenocytes pre-incubated with H_2_O_2_ 100 and 200 µM for 3 h and afterwards exposed to loading concentrations of Meloxicam, Cmp **2**, and Cmp **7** for 24 h. Values are normalized on 3-(4,5-dimethylthiazol-2-yl)-2,5-diphenyltetrazolium bromide (MTT) data obtained from the same experiment. DMSO = cultures pre-incubated with H_2_O_2_ and treated with DMSO 0.2% (vehicle). a = *p* < 0.01; b = *p* < 0.001; c = *p* < 0.0001; between samples and DMSO alone; d = *p* < 0.01; e = *p* < 0.001; f = *p* < 0.0001 between samples and Meloxicam at the same concentration.

After having observed a counteraction of the H_2_O_2_ effect in terms of augmented cell metabolism in the three donors and having showed their wide variability, cytotoxicity occurrence was investigated in the donor pool after the administration of CAI-CORM hybrids for 24 h (Figure 3).

Our group has already reported that rotator cuff-derived tenocytes respond to H_2_O_2_ increasing LDH release dramatically, already after short exposure times [5]. As expected, cells pre-incubated with 100 or 200 µM H_2_O_2_ release LDH in a great extent (21.6% and 37.9%, respectively). Meloxicam seems weakly effective in the counteraction of cytotoxicity, especially after the 100 µM H_2_O_2_ pre-incubation. On the other hand, LDH release is decreased in the presence of Cmp **2** and even more lowered with Cmp **7**. More in details, Cmp **2** is the best compound in counteracting LDH leakage after the 100 µM H_2_O_2_ pre-incubation (15.9% at 25 µM), whereas Cmp **7** starts to be effective only after a dose of 50 µM. However, Cmp **7** rapidly and significantly decreases lactate released from tenocytes pre-incubated with 200 µM H_2_O_2_ up to 16.7% at 25 µM. Nevertheless, Cmp **2** has a comparable effect at 50 µM.

The spread of cell metabolism when compounds **2** and **7** were administered without H_2_O_2_ is absent when tenocytes are exposed to Meloxicam alone. Moreover, both the compounds are capable of better counteracting H_2_O_2_-induced cytotoxicity in terms of cell metabolism and LDH release mainly at 25 and 50 µM compared to Meloxicam. Besides dual CAI-CORM hybrids are more effective in the reversal of cytotoxicity with the 200 µM H_2_O_2_ pre-incubation, the LDH percentage in the presence of DMSO alone (vehicle) after 24 h is extremely high. It is therefore plausible to assume that cells are in a pre-necrotic condition where other molecular mechanisms could be involved beyond oxidative stress [26]. Thus, only the sub-toxic concentration of 100 µM H_2_O_2_ and doses of 25 and 50 µM were used for further analyses.

### 3.4. Compounds ***2*** and ***7*** Increase the Entrance in the G2 Phase of Tenocytes under Oxidative Stress Conditions

**Figure 4:**
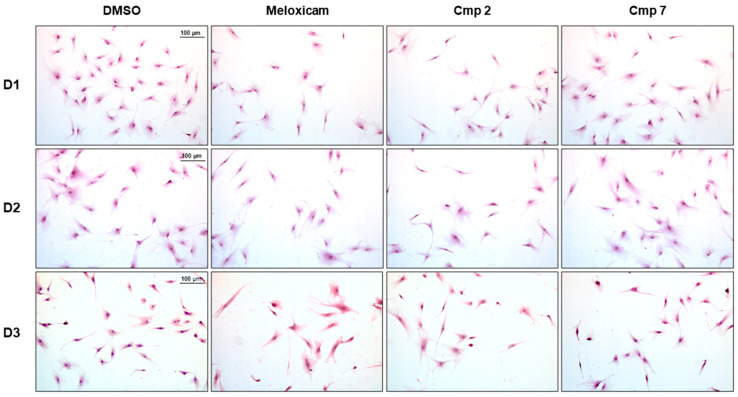
Hematoxylin/eosin (H&E) staining of tendon-derived cells pre-incubated with hydrogen peroxide (H_2_O_2_) in the presence of Meloxicam and Cmp **2** and Cmp **7**. Light microscopy images show tenocytes isolated from the three donors (D1 = donor 1; D2 = donor 2; D3 = donor 3), pre-incubated with H_2_O_2_ 100 µM for 3 h and afterwards exposed to Meloxicam, Cmp **2**, and Cmp **7** 50 µM for 24 h, stained with H&E. Magnification 100×. Bar scale represents 1 cm = 100 µm.

Since an augmented metabolism is tightly related to cell proliferation and functions, tenocytes pre-incubated with 100 µM H_2_O_2_ and consequently exposed to compounds **2** and **7** up to 50 µM underwent cell cycle analysis after 72 h of exposure. A morphological observation of tenocytes from the three donors pre-incubated with 100 µM H_2_O_2_ and exposed to compounds at 50 µM for 24 h after haematoxylin/eosin staining (Figure 4), revealed an increased number of cells in the presence of Cmp **7** with respect to Cmp **2**. Likewise, the bicinchoninic acid (BCA) assay measured a spread in terms of the protein amount with Cmp **7** after 24 h compared to Cmp **2**, Meloxicam, and DMSO alone (546.6 µg/mL), confirming an augmented metabolism. Indeed, the concentration of protein is assessed at 918.9 µg/mL already with Cmp **7** at 6.25 µM and around 760 µg/mL with 25 and 50 µM (SI-Figure S3).

**Figure 5:**
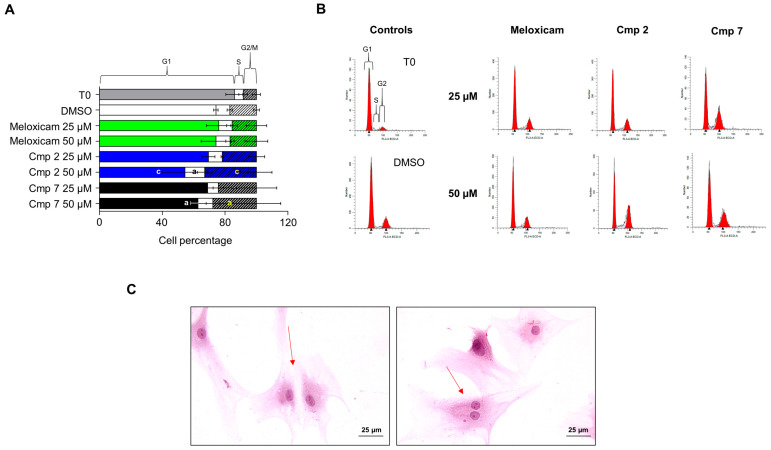
Cell cycle analyzed in tendon-derived cells pre-incubated with hydrogen peroxide (H_2_O_2_) in the presence of Meloxicam and Cmp **2** and Cmp **7**. Data are reported as means ± standard deviations of independent experiments with the three donors pooled (n = 9). (**A**) The bar graph shows cell percentages in the various phases of cell cycle (G1, S, and G2) of tenocytes pre-incubated with H_2_O_2_ 100 µM for 3 h and afterwards exposed to Meloxicam, Cmp **2**, and Cmp **7** at 25 and 50 µM for 72 h. DMSO = cultures pre-incubated with H_2_O_2_ and treated with DMSO 0.2% (vehicle). a = *p* < 0.01; b = *p* < 0.001; c = *p* < 0.0001 between samples and DMSO alone. (**B**) Cell cycle profiles represented by fluorescence emission peaks obtained after the propidium iodide staining (*y*-axis = cell count; *x*-axis = propidium iodide fluorescence emission in the FL- channel). (**C**) Hematoxylin/eosin staining of tenocytes pre-incubated with 100 µM H_2_O_2_ for 3 h and exposed to Cmp **7** 25 µM for 72 h. Magnification 400x. Bar scale represents 1 cm = 25 µm. Red arrows indicate cells in mitosis.

As for cell cycle analysis (Figure 5), profiles of tenocytes in the presence of Meloxicam and CAI-CORM hybrids were compared to T0, which represents proliferating cells from routine culture, and tenocytes pre-incubated with H_2_O_2_ in the presence of DMSO alone (Figure 5A,B). As expected, tenocytes in the DMSO control display an increased G2 phase compared to the one of T0 (16.9% and 8.44%, respectively). It is well known indeed that in response to overuse or inflammation, tenocytes increase their metabolic activity and multiply [6,27]. In the presence of Meloxicam, percentages of cells in the various phases of cell cycle are comparable to the ones of the DMSO control. When Cmp **2** is present, a dose-dependent increase in terms of cell percentage in the G2 phase is registered, being doubled with respect to DMSO alone at 50 µM (32.9%). As well, tenocytes in the G2 phase after the administration of Cmp **7** are increased already at 25 µM (24.2%) with respect to Cmp **2** at the same concentration (21.7%). Cells presumably undergoing mitosis in the presence of Cmp **7** at 25 µM are observed also microscopically, as shown in Figure 5C by red arrows.

### 3.5. Compound ***2*** Enhances Collagen Type I Secretion in H_2_O_2_-Stimulated Tenocytes

**Figure 6:**
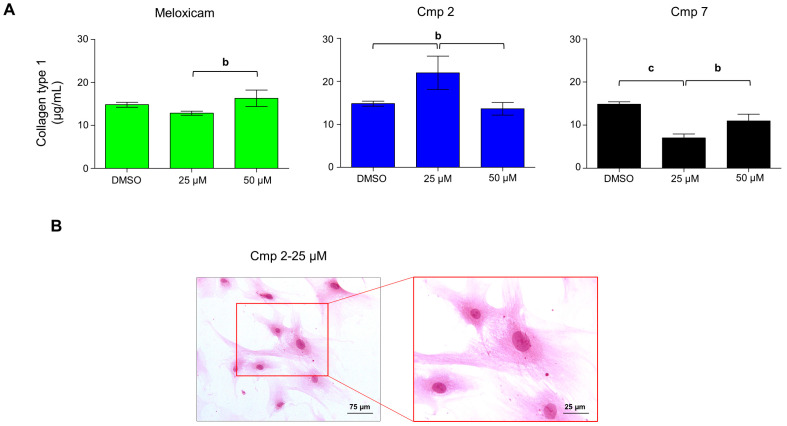
Collagen type I secretion from tendon-derived cells pre-incubated with hydrogen peroxide (H_2_O_2_) in the presence of Meloxicam and Cmp **2** and Cmp **7**. Data are reported as means ± standard deviations of independent experiments with the three donors pooled (n = 9). (**A**) Bar graphs show collagen type I concentration obtained by ELISA assay in cell supernatants (µg/mL) of tenocytes pre-incubated with H_2_O_2_ 100 µM for 3 h and afterwards exposed to Meloxicam, Cmp **2**, and Cmp **7** at 25 and 50 µM for 72 h. Values are normalized on MTT data from the same experiment. DMSO = cultures pre-incubated with H_2_O_2_ and treated with DMSO 0.2% (vehicle). b = *p* < 0.001 and c = *p* < 0.0001 between samples and DMSO alone. (**B**) Hematoxylin/eosin staining of tenocytes pre-incubated with 100 µM H_2_O_2_ for 3 h and exposed to Cmp **2** at 25 µM for 72 h. Magnification 200× and 400×. Bar scale represents 1 cm = 75 and 25 µm, respectively.

**Figure 7:**
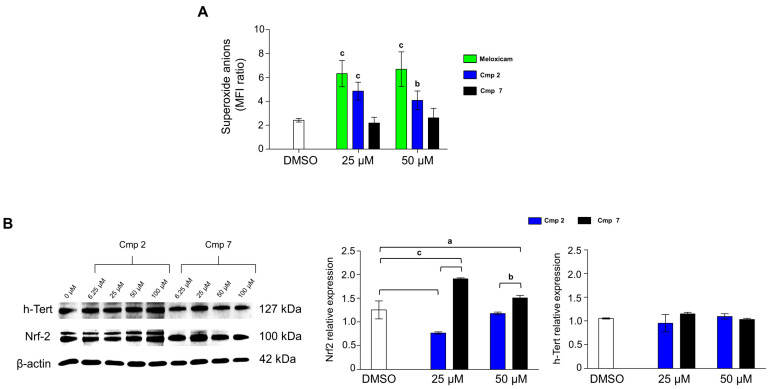
Generation of superoxide anions and Nrf2 and h-Tert expression levels in tendon-derived cells pre-incubated with hydrogen peroxide (H_2_O_2_) in the presence of Meloxicam, Cmp **2** and Cmp **7**. Data are reported as means ± standard deviations of independent experiments with the three donors pooled (n = 9). (**A**) The bar graph shows mean fluorescence intensity (MFI) ratios related to the emission in the FL-2 channel which are proportional to the generation of superoxide anions in tenocytes pre-incubated with H_2_O_2_ 100 µM for 3 h and exposed as indicated for 24 h. Values are the ratios of the MFI generated from each sample on the unstained control (negative). (**B**) Western blot analysis of Nrf2 and h-Tert protein expression in tenocytes in the same indicated experimental conditions of (**A**). β-actin is used as loading control. Bar graphs display densitometric values expressed as mean D.O.I normalized on the ones of the loading control. a = *p* < 0.01; b = *p* < 0.001; c = *p* < 0.0001 between samples and DMSO alone. DMSO = cultures pre-incubated with H_2_O_2_ and treated with DMSO 0.2%.

Distinct cytoprotective roles of CO have been recently elucidated as well as its metabolic effects in terms of ATP production and augmented cell viability under pro-inflammatory substances like LPS [28]. Increasing cell metabolism and proliferation are particularly important for tendon tissue repair after the acute inflammatory phase. As a matter of fact, tendon healing occurs in different stages, among which the consolidation phase involves both high cell metabolism and collagen type I secretion [27]. In this light, collagen type I concentration was quantified in cell supernatants of tenocytes in the same conditions of cell cycle analyses (Figure 6). Surprisingly, collagen type I amount is dramatically increased with Cmp **2** at 25 µM (22 µg/mL) with respect to the DMSO control (14.8 µg/mL) and compared to Meloxicam and Cmp **7**. In that experimental condition, microscopic observations reveal the presence of a network of flattened cell extensions between adjacent cells which have been proven to be critical structures that enable matrix remodeling in fibroblasts secreting collagen fibers [29] (Figure 6B). Being less efficient as regards the CO releasing profile compared to that of Cmp **7** [17] and in counteracting H_2_O_2_-related cytotoxicity, Cmp **2** is instead extremely active in increasing collagen type I secretion, as if tenocytes were in a different stage of the healing. For better understanding molecular mechanisms underlying these observations, superoxide anions generation and Nrf2 expression levels were quantified to verify the modulation of the redox equilibrium whereas h-Tert was analyzed as a marker of senescence (Figure 7).

### 3.6. Compound ***7*** Triggers Nrf2 Expression and Acts as a Superoxide Scavenger

Our previous work reported the involvement of the transcription factor Nrf2 in the counteraction of H_2_O_2_-induced cytotoxicity in tenocytes [5], while h-Tert expression has been related to tendon development [30]. In the present experimental model, the MitoSox staining reveals that Cmp **7** acts as a potent superoxide scavenger compared to Meloxicam and Cmp **2** (Figure 7A). More in details, the superoxide amount with Cmp **7** at 25 µM is 2.1 folds of the negative control (equal to 1, not shown) and 2.6 at 50 µM. It should be noted that Cmp **7** is the only compound which shows values comparable to the one of the DMSO control (2.4 folds). Meloxicam fails in decreasing superoxide anion concentrations even at the highest dose administered of 50 µM (6.7 folds), while Cmp **2** is more effective than Meloxicam but less than Cmp **7** (4.1 folds). The ineffectiveness of Meloxicam can be ascribed to its inhibitory activity on superoxide dismutase (SOD), which can cause glutathione depletion [31] and can be responsible for the debated use of NSAIDs in tendinopathies. Since Meloxicam mechanism of action is broadly known acting as a preferential COX2 potent inhibitor [20], molecular analyses at the protein level were performed only on compounds **2** and **7**.

As expected, Nrf2 is expressed in tenocytes after the H_2_O_2_ pre-incubation (Figure 7B). In accordance with the superoxide anion decreased production in the presence of Cmp **7**, Nrf2 expression levels are dramatically increased both at 25 and 50 µM as a sign of activated antioxidant cell response in tenocytes [5]. In addition to the better CO release rate with respect to Cmp **2**, Cmp **7** is a coumarin-derivative and the protective effect of coumarins against oxidative stress through Nrf2 activation is well established [32]. As for h-Tert is very well expressed in all the experimental conditions, meaning that tendon-derived cells still preserve their proliferation potential, and they are not terminally differentiated into post-mitotic cells [30].

**Figure 8:**
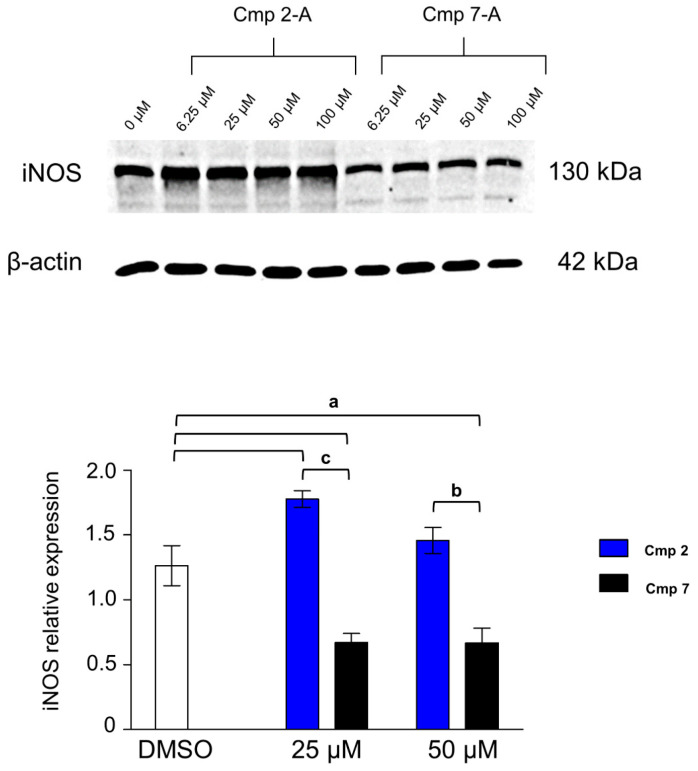
Inducible nitric oxide synthase (iNOS) expression levels in tendon-derived cells pre-incubated with hydrogen peroxide (H_2_O_2_) in the presence of Cmp **2** and Cmp **7**. Data are reported as means ± standard deviations of independent experiments with the three donors pooled (n = 9). Western blot analysis of the iNOS expression in tenocytes pre-incubated with H_2_O_2_ 100 µM for 3 h and exposed as indicated for 24 h. β-actin is used as a loading control. The bar graph displays densitometric values expressed as mean DOI normalized on the ones of the loading control. a = *p* < 0.01; b = *p* < 0.001; c = *p* < 0.0001 between samples and DMSO alone. DMSO = cultures pre-incubated with H_2_O_2_ and treated with DMSO 0.2%.

The analyses of molecules related to the redox equilibrium and to senescence have partly clarified the molecular mechanisms underlying the biological observations obtained with compounds **2** and **7**. It is broadly known that nitric oxide (NO) plays a relevant role in repairing an injured tendon [33] and our previous work reported a role of inducible nitric oxide synthase (iNOS) in tenocytes under oxidative stress conditions [4]. Thus, expression levels of iNOS were quantified in the presence of compounds **2** and **7** (Figure 8).

### 3.7. iNOS Is Differentially Expressed in the Presence of Compounds ***2*** and **7**

Western blot analysis reveals a robust iNOS expression in the DMSO control, accordingly to our previous results [4]. Intriguingly, the presence of Cmp **2** dramatically raises iNOS expression whereas it is only weakly expressed in the presence of Cmp **7**. It has been demonstrated that NO favors tendon healing at the molecular level by increasing collagen synthesis after 7 days of treatment in an in vitro tendon cell culture [34]. The enhanced expression of collagen type I in the presence of Cmp **2** (Figure 6A) can be therefore ascribed to the overproduction of nitric oxide through iNOS, an event which has been described as fundamental in triggering extracellular matrix-related genes, such as collagens I, II, and IV [35]. It should be also noted that Cmp **2** acts as a human CA12 inhibitor in vitro (*K*_i_ = 3.46 µM) [17] and the interactome of this isoform has been linked to collagen degradation through metalloproteinase 14 (MMP-14) [36]. The involvement of MMP-14 and its interplay with collagen production has been already reported for rotator cuff-derived cells [4] and further analyses are required to clarify these speculations.

### 3.8. NF-kB Nuclear Translocation Is Enhanced in the Presence of Compound ***2***

**Figure 9:**
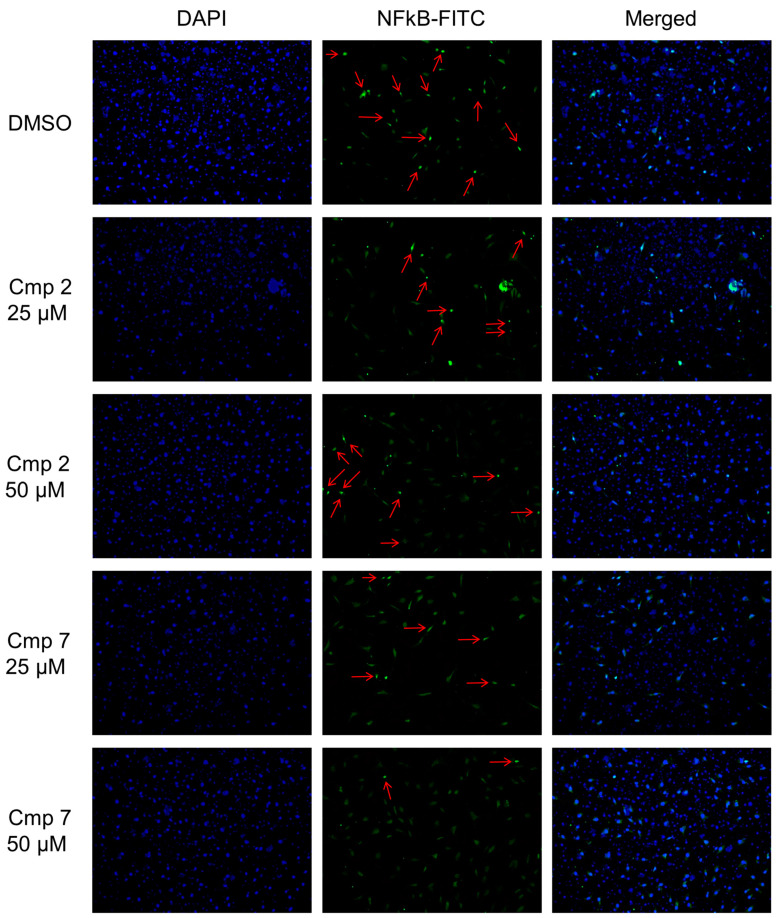
NF-kB expression in tendon-derived cells pre-incubated with hydrogen peroxide (H_2_O_2_) in the presence of Cmp **2** and Cmp **7**. Representative images from three independent experiments of the expression of NF-kB after immunofluorescence staining in tenocytes pre-incubated with H_2_O_2_ 100 µM for 3 h and exposed as indicated for 24 h. NF-kB is stained in green (Fluorescein Isothiocyanate, FITC) and cell nuclei are highlighted in blue (4′,6-diamidino-2-phenylindole, DAPI). Red arrows indicate nuclear NF-kB.

The formation of NO under pro-inflammatory conditions is mostly a result of NF-kB enhanced nuclear translocation which regulates expression of NOX2 and iNOS enzymes [37]. To confirm iNOS expression data, the immunofluorescence of NF-kB was performed in the presence of compounds **2** and **7** in the same conditions of protein analyses (Figure 9). Notably, NF-kB shows a nuclear localization in the presence of Cmp **2**, while it is mostly cytosolic after the Cmp **7** administration. More in details, the number of positive nuclei significantly decrease in the presence of Cmp **7** 50 µM (3 nuclei) compared to Cmp **2** at the same concentration (11 nuclei). Furthermore, the intensity of Nf-kB cytosolic fluorescence results clearly increased with Cmp **7** at the highest concentration, resembling the elongated fibroblast-like morphology of tenocytes. The interplay between NF-kB and Nrf2 in cell responses towards oxidative stress is finely regulated and it has been recently well elucidated [38,39]. Expression of HO-1 as an antioxidant protein is controlled by both NF-kB and Nrf2 and links the activities of both transcription factors. It has been therefore reported that Nrf2-induced expression of HO-1 inhibits NF-kB activity, indicating its anti-inflammatory function [37]. It is plausible to assume that Cmp **7** acts as a canonical coumarin-based compound and activates Nrf2-related cell responses towards oxidative stress while Cmp **2** acts on the counterpart NF-kB.

## 4. Conclusions

Five dual CAI-CORM hybrids, previously evaluated in LPS-stimulated macrophages, have been further investigated for their role in the counteraction of oxidative stress in human rotator cuff-derived tenocytes under H_2_O_2_ stimulation. From our results, it can be therefore suggested that both compounds counteract oxidative stress-related cytotoxicity through CO release and thus directly stimulating HO-1-related antioxidant responses, as already reported for macrophages in our previous work [17]. The coumarin-derivative Cmp **7** activates Nrf2, which plausibly transcribes for additional HO-1 and SOD, decreases superoxide production, and counteracts H_2_O_2_-induced oxidative stress. Moreover, Cmp **7** could own a dual function because it inhibits human CA9 in vitro which can be involved in tendon injuries. On the other hand, Cmp **2** activates NF-kB which transcribes for iNOS, an event that probably produces massive amounts of NO and is involved in collagen type 1 production. Moreover, acting as a potential CA12 inhibitor in vitro, the function of Cmp **2** can be partly ascribed to this CA interactome. Moreover, Cmp **2** is extremely effective in terms of collagen type 1 deposition, a crucial event for tendon healing after the counteraction of inflammation.

Taken together, these results lay the grounds for further molecular studies on the involvement of CA inhibition in the inflamed tendon and on the use of CAI-CORM hybrids in a clinical situation.

## Data Availability

Data are contained within the article.

## Supplementary Materials

The following are available online at https://www.mdpi.com/2227-9059/9/2/141/s1. Supplementary Materials-Figure S1. Metabolic activity of tendon-derived cells in the presence of hydrogen peroxide and Meloxicam. Data are reported as means ± standard deviations of independent experiments with the three donors pooled (n = 9). (**A**) The bar graph represents the cell dose-response towards loading concentrations of hydrogen peroxide (0–800 µM) after 3 and 24 h. Untreated cultures (i.e., only complete medium) were set as control (100% of the metabolic activity). a= *p* < 0.01 and c= *p* < 0.0001 between samples and the untreated control. (**B**) The bar graph represents the cell dose-response towards loading concentrations of Meloxicam (0–200 µM) after 24 and 72 h. Tenocytes in the presence of 0.2% of DMSO (vehicle) were set as control. Supplementary Materials-Figure S2. Metabolic activity of tendon-derived cells in the presence of CAI-CORM hybrids. Data are reported as means ± standard deviations of independent experiments with the three donors separately (n = 9). D1 = donor 1; D2 = donor 2; D3 = donor 3. Bar graphs show the cell dose-response towards loading concentrations of compounds **1**, **2**, **6**, **7**, and **8** (0–200 µM) after 24 and 72 h. Tenocytes in the presence of 0.2% of DMSO (vehicle) were set as control (100% of the metabolic activity). a = *p* < 0.01; b = *p* < 0.001; c = *p* < 0.0001 between samples and DMSO alone. Supplementary Materials-Figure S3. Total amount of protein in tendon-derived cells pre-incubated with hydrogen peroxide (H_2_O_2_) in the presence of Meloxicam and Cmp **2** and Cmp **7**. Data are reported as means ± standard deviations of independent experiments with the three donors pooled (n = 9). Bar graphs represent the protein concentration (µg/mL) measured by the BCA assay in tenocytes pre-incubated with H_2_O_2_ 100 and 200 µM for 3 h and afterwards treated with Meloxicam and compounds **2** (Cmp **2**) and **7** (Cmp **7**) (0–100 µM, where the 0 µM is named after DMSO) for 24 h. DMSO = cultures pre-incubated with H_2_O_2_ and treated with DMSO 0.2%. a = *p* < 0.01; b = *p* < 0.001; c = *p* < 0.0001 between samples and DMSO alone; d = *p* < 0.01; e = *p* < 0.001; f = *p* < 0.0001 between samples and Meloxicam at the same concentration; g = *p* < 0.01; h = *p* < 0.001; i = *p* < 0.0001 between Cmp **2** and Cmp **7** at the same concentration.
