# Supplementary material for: Dual Acting Carbon Monoxide Releasing Molecules and Carbonic Anhydrase Inhibitors Differentially Modulate Inflammation in Human Tenocytes
Source: Biomedicines. 2021 Feb 1;9(2):141. doi: 10.3390/biomedicines9020141 (PMC7912830; doi:10.3390/biomedicines9020141)
Supplement: Supplementary file 1 [file biomedicines-09-00141-s001.pdf]

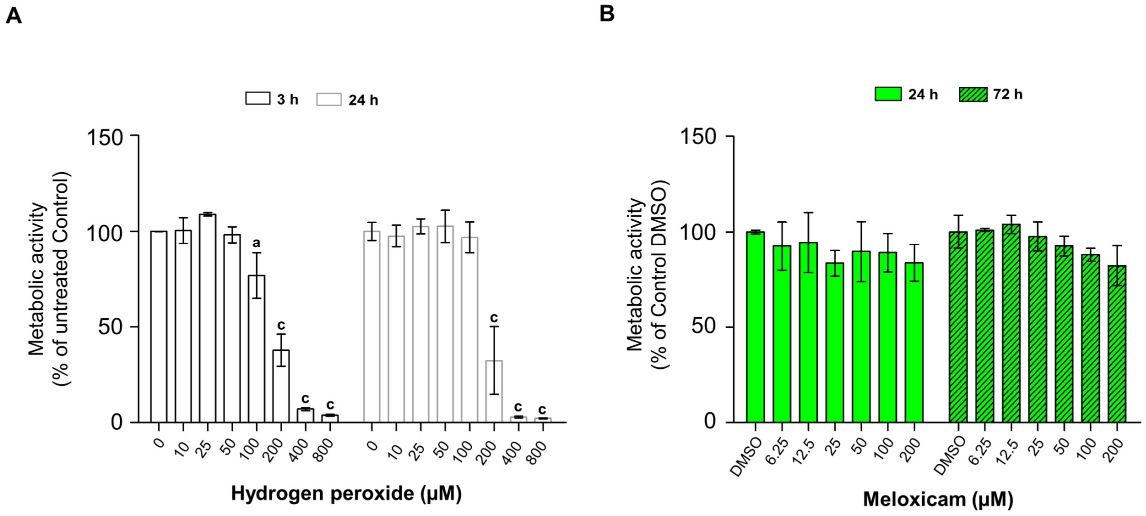

Figure 1. Metabolic activity of tendon-derived cells in the presence of hydrogen peroxide and Meloxicam. Data are reported as means ± standard deviations of independent experiments with the three donors pooled (n=9). (**A**) The bar graph represents the cell dose-response towards loading concentrations of hydrogen peroxide (0-800 µM) after 3 and 24 h. Untreated cultures (i.e., only complete medium) were set as control (100% of the metabolic activity). a= p<0.01 and c= p<0.0001 between samples and the untreated control. (**B**) The bar graph represents the cell dose-response towards loading concentrations of Meloxicam (0-200 µM) after 24 and 72 h. Tenocytes in the presence of 0.2% of DMSO (vehicle) were set as control.

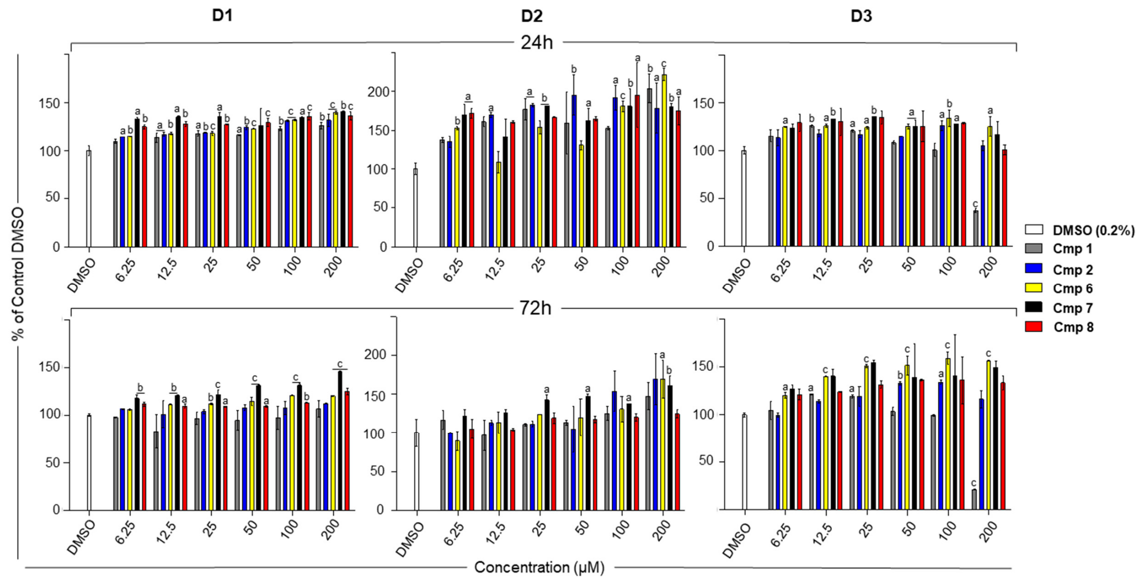

Figure S2. Metabolic activity of tendon-derived cells in the presence of CAI-CORM hybrids. Data are reported as means ± standard deviations of independent experiments with the three donors separately (n=9). D1= donor 1; D2 = donor 2; D3 = donor 3. Bar graphs show the cell dose-response towards loading concentrations of compounds **1**, **2**, **6**, **7** and **8** (0-200 μM) after 24 and 72 h. Tenocytes in the presence of 0.2% of DMSO (vehicle) were set as control (100% of the metabolic activity). a = $p<0.01$; b = $p<0.001$; c = $p<0.0001$ between samples and DMSO alone.

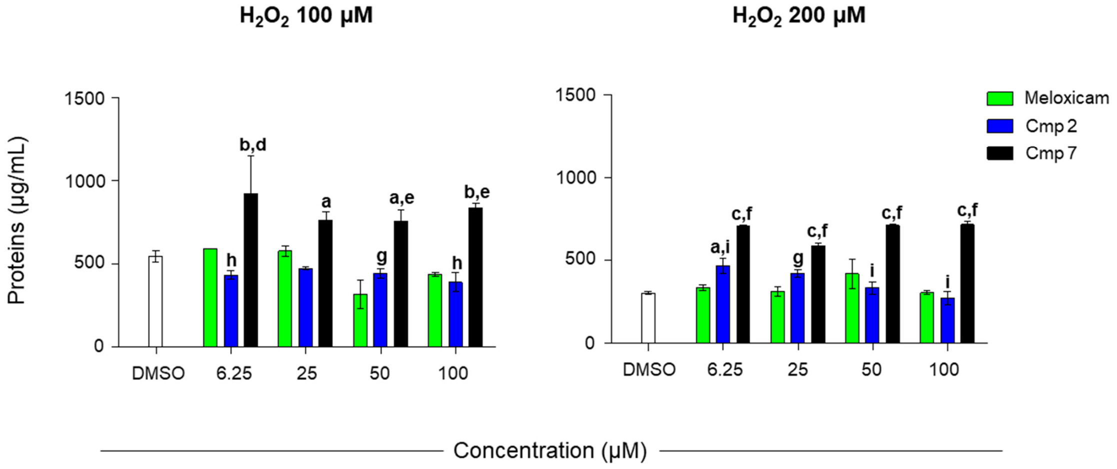

Figure S3. Total amount of protein in tendon-derived cells pre-incubated with hydrogen peroxide (H_2O_2) in the presence of Meloxicam and Cmp 2 and Cmp 7. Data are reported as means ± standard deviations of independent experiments with the three donors pooled (n=9). Bar graphs represent the protein concentration (μg/mL) measured by the BCA assay in tenocytes pre-incubated with H_2O_2 100 and 200 μM for 3 h and afterwards treated with Meloxicam and compounds **2** (Cmp **2**) and **7** (Cmp **7**) (0-100 μM, where the 0 μM is named after DMSO) for 24 h. DMSO = cultures pre-incubated with H_2O_2 and treated with DMSO 0.2%. a = $p<0.01$; b = $p<0.001$; c = $p<0.0001$ between samples and DMSO alone; d = $p<0.01$; e = $p<0.001$; f = $p<0.0001$ between samples and Meloxicam at the same concentration; g = $p<0.01$; h = $p<0.001$; i = $p<0.0001$ between Cmp **2** and Cmp **7** at the same concentration.
